# Supervised Wavelet Method to Predict Patient Survival from Gene Expression Data

**DOI:** 10.1155/2014/618412

**Published:** 2014-11-03

**Authors:** Maryam Farhadian, Paulo J. G. Lisboa, Abbas Moghimbeigi, Jalal Poorolajal, Hossein Mahjub

**Affiliations:** ^1^Department of Epidemiology & Biostatistics, School of Public Health, Hamadan University of Medical Sciences, Hamadan, Iran; ^2^School of Computing and Mathematical Sciences, Liverpool John Moores University, UK; ^3^Modeling of Noncommunicable Disease Research Center, Department of Biostatistics and Epidemiology, School of Public Health, Hamadan University of Medical Sciences, Hamadan, Iran; ^4^Research Center for Health Sciences and Department of Biostatistics and Epidemiology, School of Public Health, Hamadan University of Medical Sciences, Hamadan, Iran

## Abstract

In microarray studies, the number of samples is relatively small compared to the number of genes per sample. An important aspect of microarray studies is the prediction of patient survival based on their gene expression profile. This naturally calls for the use of a dimension reduction procedure together with the survival prediction model. In this study, a new method based on combining wavelet approximation coefficients and Cox regression was presented. The proposed method was compared with supervised principal component and supervised partial least squares methods. The different fitted Cox models based on supervised wavelet approximation coefficients, the top number of supervised principal components, and partial least squares components were applied to the data. The results showed that the prediction performance of the Cox model based on supervised wavelet feature extraction was superior to the supervised principal components and partial least squares components. The results suggested the possibility of developing new tools based on wavelets for the dimensionally reduction of microarray data sets in the context of survival analysis.

## 1. Introduction

Microarray studies are widely used in biological and medical studies because they allow researchers to monitor tens of thousands of gene expression profiles simultaneously. Much of the interest in microarray data analysis derives from the potential of identifying the genes that relate to biological processes, the classification of tumor types, the stages based on gene expression patterns, and the study of gene interactions [1, 2]. However, because microarray data sometimes include patients survival data, it is important to study patients survival times (response) in terms of their corresponding gene expression levels (predictors). The discovery of the relationship between time to event (survival time) and gene expression profiles as covariates provides the possibility to obtain more accurate diagnosis and advanced treatment [3]. It is estimated that high-dimensional gene expression data could noticeably enhance the predictive ability of such survival models [4].

Survival analysis is a statistical method that especially deals with the modeling and analysis time from a well-defined time origin until the occurrence of some event or end point of interest. A major complexity of analyzing such data is right censoring, where the event of interest is known to occur only after a certain time point. One popular regression model that takes into account the censored response is the Cox Proportional Hazards (CPH) regression model [5]. A substantial challenge in this setting comes from the fact that the number of genomic variables *p* is usually much larger than the number of subjects *n* (i.e., *p* ≫ *n*). Existing statistical methods such as CPH model require fewer predictors than cases [4]. Thus, a crucial step towards the application of microarrays in survival prediction is the dimensionality reduction from the gene expression profiles. In recent years, both feature selection and feature extraction methods have been widely used to predict the survival of cancer patients based on gene expression data [6].

Rosenwald et al. described a feature selection approach for identifying genes related to survival time that fits CPH models to each gene and selected those that pass a threshold for significance [7]. Liu et al. presented the adaptive *L*_1/2_ shooting regularization method, which is used for variable selection in the CPH model [8]. Alizadeh et al. described an approach in which they first clustered the genes and then fitted a CPH model using the average expression level of each cluster as a covariate [1]. Nguyen and Rocke and Park et al. considered the problem of relating survival time to gene expression by reducing the dimensionality via partial least squares method. The first few linear combinations of gene expressions obtained via PLS were subsequently used in a CPH regression model for predicting the survival probabilities [9, 10]. Li and Luan developed a penalized estimation procedure for the CPH model using kernels, under the assumption that the covariate effects were smooth functions of gene expression levels [11].

Several studies have compared dimension reduction methods in survival prediction based on microarray data. Bøvelstad et al. applied seven dimension reduction methods in order to predict survival in patients with diffuse large B-cell lymphoma (DLBCL) using gene expression dataset. Totally, their results showed that the ridge regression had best performance [4].

One of the methods used for feature extraction from the high dimensional data is wavelet transform. Normally, one dimensional discrete wavelet transform (DWT) is used to reduce dimensionality in the analysis of high dimensional biomedical data [12]. The primary intuition for applying wavelets in the case of gene expression is that genes are often coexpressed in groups. It would be useful to treat the group as a single variable, akin to the motivation behind methods such as principal component analysis [12]. Studies showed that this method has acceptable performance in the field of dimension reduction in the classification framework [13–16].

However, few studies have used wavelet transform in the area of survival analysis. For example, Liu et al. used continuous wavelet transform combined with a genetic algorithm to select genes related to survival in colon cancer [15]. This study aimed to introduce a dimension reduction strategy for transforming the high-dimensional gene expression data into a low dimensional space based on wavelet transform. Accordingly, a predictive survival model was built upon the reduced dimensional space. Then, the proposed novel supervised method of feature extraction was compared with the supervised principal component analysis (PCA) and the supervised partial least squares (PLS) method.

## 2. Material and Methods

### 2.1. Simulation Setup

We performed simulation study to evaluate and compare the performance of the proposed supervised wavelet method with supervised PCA and supervised PLS. The simulated data set was first presented by Bair and Tibshirani, for evaluation purposes [17]. Following Bair and Tibshirani, simulated data set X consisted of 5000 genes and 100 samples. All expression values were generated as standard normal random numbers with a few exceptions. Genes 1–50 in samples 1–50 had a mean of 1.0. We randomly selected 40% of the samples to have a mean of 2.0 in genes 51–100, 50% of the samples to have a mean of 1.0 in genes 101–200, and 70% of the samples to have a mean of 0.5 in genes 201–300.

The survival times of samples 1–50 were generated as normal random numbers with a mean of 10.0 and a standard deviation of 2.0, and the survival times of samples 51–100 were generated as normal random numbers with a mean of 8.0 and a standard deviation of 3.0. For each sample, a censoring time was generated as a normal random number with a mean of 10.0 and a standard deviation of 3.0. If the censoring time turned out to be less than the survival time, the observation was considered to be censored [17].

### 2.2. Real-Life Datasets

We applied the supervised wavelet transform method to a set of gene expression data with survival information on two real datasets. The first dataset was related to the diffuse large B-cell lymphoma (DLBCL) dataset of Rosenwald et al. and the second dataset was related to the lung cancer dataset of Beer et al. [7, 18].

The DLBCL dataset included expression measurements of 7,399 genes on 240 patients, together with their survival times. A total of 138 deaths were observed during the study with the median death time of 2.8 years. The dataset is available at http://llmpp.nih.gov/lymphoma/data.shtml.

The lung cancer dataset also included expression measurements of 7,129 genes on 86 lung adenocarcinoma patients, together with their survival times. The survival times were observed in 24 patients and the censored times in 62 patients. A detailed description of lung cancer dataset can be found in the original publication [18]. We used the dataset from the study conducted by Zhao and Simon in 2008 [19].

### 2.3. Cox Proportional Hazards Model

The CPH model is the most commonly used model in survival analysis. It is also known as the Cox regression model. It factorizes the time dependence of the event rate from the covariate dependence as follows:

(1) $$\begin{array}{c} h\left(t,x\right)=h_{0}\left(t\right)\exp\left(\beta^{T}x\right), \end{array}$$

where *h*(*t*, *x*) represents the hazard function at time *t* for a subject with covariates *x*. For different covariates, CPH regression models the hazard as a proportional factor applied to time-dependent baseline hazard that corresponds to a reference population for which the covariate values are all zero. This baseline hazard function is *h*_0_(*t*) and the effect of the covariates *x* is modeled linearly using *β* ^*T*^ *x*, which is known as the risk score. The coefficient vector *β* is estimated by maximizing the partial likelihood:

(2) $$\begin{array}{c} l\left(\beta \right)=\overset{k}{\underset{j=1}{\prod }}\left(\frac{\exp\left(\beta^{T}x_{j}\right)}{\sum_{l\in R_{j}}\exp\left(\beta^{T}x_{l}\right)}\right), \end{array}$$

where *R*_*j*_ represents all patients at risk at the *j*th failure time and *k* is the number of distinct failure times. The hazard ratio between different observations *i* and *j* by (1) is assumed to be constant and independent of time:

(3) $$\begin{array}{c} \frac{h_{i}\left(t,x_{i}\right)}{h_{j}\left(t,x_{j}\right)}=\frac{\exp\left(\beta^{T}x_{i}\right)}{\exp\left(\beta^{T}x_{j}\right)}. \end{array}$$

Consequently, the Cox regression model is a proportional hazards model [5].

### 2.4. Wavelet Transform

A wavelet is a “small wave,” which has its energy concentrated in time. In signal processing, a transformation technique is used to transfer a data in another domain where hidden information can be extracted. Wavelets have a nice feature of local description and separation of signal characteristics and give a tool for the analysis of transient or time-varying signal [12]. A wavelet is a set of orthonormal basis functions generated from dilation and translation of a single scaling function or father wavelet (*φ*) and a mother wavelet (*ψ*).

Wavelet transforms are classified into two different categories: the continuous wavelet transforms (CWT) and the discrete wavelet transforms (DWT). DWT is a linear operation that operates on a data vector, transforming it into a wavelet's coefficient. The idea underlying DWT is to express any function *f*(*t*) ∈ *L* ^2^(*R*) in terms of *φ*(*t*) and *ψ*(*t*) as follows:

(4) ft=∑kc0kφt−k +∑k ∑j=1djk2−j/2ψ2−jt−k=∑kcj0k2−j0/2φ2−j0t−k +∑k ∑j=j0djk2−j/2ψ2−jt−k,

where *φ*(*t*), *ψ*(*t*), *c*_0_, and *d*_*j*_ represent the scaling function, mother wavelet function, scaling coefficients (approximation coefficients) at scale 0, and detail coefficients at scale *j*, respectively. The variable* k *is the translation coefficient for the localization of gene expression data. The scales denote the different (low to high) scale bands. The variable symbol *j*_0_ is scale (level) number selected.

One-dimensional discrete wavelet transform decomposes a signal as a sum of wavelets at different time shifts and scales (frequencies) using DWT. For this purpose, the signal is passed through series of high pass and low pass filters in order to analyze low as well as high frequencies in the signal as follows:

(5) $$\begin{array}{c} c_{j+1}=\underset{m}{\sum }h\left(m-2k\right)c_{j}\left(m\right),\\ d_{j+1}=\underset{m}{\sum }h_{1}\left(m-2k\right)c_{j}\left(m\right), \end{array}$$

where *h*(*m* − 2*k*) and *h*_1_(*m* − 2*k*) are the low-pass filters and high-pass filters.

**Figure 1:**
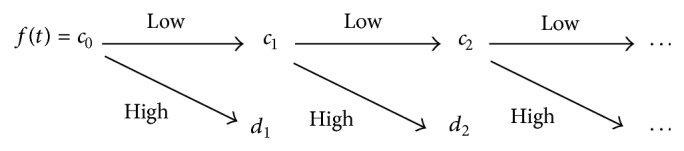
The 1D wavelet decomposition process.

The whole process of obtaining the wavelet transform of *f*(*t*) using the pyramid algorithm is shown in Figure 1.

At each level, the high pass filter produces detail coefficients (wavelet coefficients) *d*_1_, while the low pass filter associated with scaling function produces approximation coefficient (scaling coefficients) *c*_1_. Then the approximation coefficients *c*_1_ are split into two parts by using the same algorithm and are replaced by *c*_2_ and *d*_2_, and so on. This decomposition process is repeated until the required level is reached. The coefficient vectors are produced by down sampling and are only half the length of the signal or the coefficient vector at the previous level.

The main advantage of the wavelet transform is that each basis function is localized jointly in both the time and frequency domains. From a viewpoint of time-frequency, the approximation coefficients are corresponding to the larger-scale low-frequency components and the detail coefficients are corresponding to the small-scale high-frequency components. Generally, the former can be used to approximate the original signal and the latter represents some local details of the original signal [12–14, 20].

There are different families of wavelets symlet, coiflet, daubechies, and biorthogonal wavelets. They vary in various basic properties of wavelets, like compactness. Among them, Haar wavelets belonging to Daubechies wavelet family are the most commonly used wavelets in database literature because they are easy to comprehend and fast to compute.

#### 2.4.1. Supervised Wavelet Transform

The proposed method starts by adopting a univariate Cox model for each gene:

(6) $$\begin{array}{c} h\left(t,x_{g}\right)=h_{0}\left(t\right)\exp\left(\beta^{T}x_{g}\right), \end{array}$$

for each gene *g* = 1,2,…, 7399.

The covariates, each representing a different gene, are then sorted by increasing absolute values of the Wald's statistic *β*/se(*β*), which are measures of the correlation between the gene expression level and patient survival. Then, in each step, we pick out the top number of genes included with higher Wald's statistic. Then, this reduced set of genes is modeled by the one-dimensional discrete wavelet transform to extract the relevant information and finally, the wavelet approximation coefficients in the first levels of decomposition are used in a multiple Cox regression model (1). Note that numbers of selected genes in this stage are considered proportional to the sample size. The Haar wavelet transform in the first level is applied on the preselected genes.

### 2.5. Supervised Principal Components Analysis

Bair and Tibshirani and Bair et al. proposed the supervised principal components regression [17, 21]. This procedure first picks out a subset of the gene expressions that is correlated with survival by using univariate selection and then applies PCA to this subset. In our analysis, we pick out top number of genes with higher Wald's statistic. Then, we apply principal components analysis to this subset of genes and, in each step, include the top number of principal components that will be comprised of at least 75% of the total variance into a multivariate Cox model.

### 2.6. Partial Least Squares Method

Partial least squares (PLS) method is a supervised dimension reduction technique that is usually employed to correlate a response variable to the explanatory variables. PLS components are linear combinations of the predictor variables, constructed to maximize an objective criterion based on the sample covariance between response and covariates.

PLS finds components that are dependent on both the variance of the gene expressions and the covariance between the gene expressions and the survival, whereas the components in PCA only depend on the variance of the gene expressions [9]. Many methods have been suggested to perform PLS for Cox regression. We used the method which was provided by the plsRcox package. In this study, the number of PLS components was fixed like for the supervised PCA method.

### 2.7. Model Building and Model Evaluation Criteria

In order to evaluate the proposed method, in all experiments (simulation and real life), data set was randomly divided into training (2/3 of the data) and test (1/3 of the data) sets for 50 times. The methods (supervised wavelet, supervised PCA, and supervised PLS) were applied to the training set and the test set was used to calculate the evaluation measures. These data sets included 66 samples from 100 samples for simulated data, 160 samples from 240 patients for DLBCL data, and 60 samples from 86 patients for lung cancer data.

For predicting the survival of patients based on gene expression, we applied the proposed dimension reduction method, supervised PCA, and supervised PLS in stage 1 in each data set and then used the data in the reduced subspace to apply in the multiple CPH model in stage 2. In fact, following the evaluation scheme proposed by Bøvelstad et al. in each experiment, the parameters were estimated ($\hat{\beta_{\text{train}}}$) from the training data set for a given method. Then, in the test set for each patient, the obtained estimates were used to derive a prognostic index (PI) ($\text{PI}=\overset{´}{x}\hat{\beta_{\text{train}}}$). Then, this PI index was used in the Cox model for calculating the evaluation criteria. The above procedure was repeated for 50 times [3, 4]. It is noted that various numbers of preselected genes were tested in each situation. Next, the results of model evaluation criteria were computed for each dataset. These methods were compared in terms of the mean of the criteria values. MATLAB r2012a software and *R* statistical package were used for data analysis.

The predictive performance of a fitted Cox model based on supervised wavelet coefficients, supervised principal components, and supervised partial least squares components was evaluated using *R* ^2^ statistic, Concordance Probability Estimate (CPE), Likelihood ratio test statistic, Integrated Brier Score, and C index.

Moreover, in order to evaluate the effect of adding clinical information to genomic data on the performance of model for a lung cancer data set, clinical information was added to genomic data. The clinical features for each patient were included: age, sex, stage, tumor size, and nodal status.

#### 2.7.1. *R* ^2^ Statistic

*R* ^2^ statistic measures the proportion of variation in survival data that may be explained by the predictor. A predictor with good predictive performance can explain a high proportion of variation in the survival data. On the other hand, a poor predictor may explain only a little variation in the data. Accordingly, when comparing models, the model with the larger *R* ^2^ statistic is usually preferred [6]. Nagelkerke et al. suggested a general definition of the *R* ^2^ statistic that may be employed for Cox proportional hazard model as follows:

(7) $$\begin{array}{c} R^{2}=1-\exp\left(-\frac{2}{n}\left(l\left(\hat{\beta }\right)-l\left(0\right)\right)\right), \end{array}$$

where *l*(·) indicates the log-likelihood function [22]. In the present study, *R* ^2^ values are those which were provided by the coxph() *R* function.

#### 2.7.2. Concordance Probability Estimate

The discriminatory power of a statistical model is assessed by concordance probability estimate (CPE). This estimator is merely a function of the regression parameters and the covariate distribution without using the observed event and censoring times. A value of one for CPE denotes the perfect discrimination [23].

#### 2.7.3. C Index

Concordance, or C-statistic, is a valuable measure of model discrimination in analyses involving survival time data. In general, consider selecting random pairs of patients and for each pair note, whether the model correctly predicts an order, for example, a higher model score for the better result. Concordance is then the fraction of pairs for which the model is correct. A completely random prediction would have a concordance of 0.5 and a perfect rule a concordance of one [24].

#### 2.7.4. Likelihood Ratio Test Statistic

The likelihood ratio test is a global goodness-of-fit test statistic for a Cox regression model. The test statistic for the likelihood ratio test is given as follows:

(8) $$\begin{array}{c} \text{LR}=-2\ln L_{R}-\left(-2\ln L_{F}\right), \end{array}$$

where *R* denotes the reduced (PH) model obtained when all *β*'s are 0 and *F* denotes the full model. Thus, the performance is good when LR is large [5].

#### 2.7.5. Integrated Brier Score (IBS)

At a given time point *t*, the Brier score for a single subject is defined as the squared difference between observed survival status (e.g., 1 = alive at time *t* and 0 = dead at time *t*) and a model based prediction of surviving time *t*. The Brier score is given by

(9) $$\begin{array}{c} \text{BS}\left(t\right)=\frac{1}{N}\overset{n}{\underset{i=1}{\sum }}{\left(p_{i}\left(t\right)-o_{i}\left(t\right)\right)}^{2}W, \end{array}$$

where *N* is the sample size, *o*_*i*_(*t*) is the observed survival at time *t*, and *p*_*i*_(*t*) is the predicted probability at time *t*. The weight *W* is used to remove a large censoring bias. The Integrated Brier Score (IBS) is a summary of the prediction error over event time by integrating the formula (9). The smaller the Brier score is, the better the survival prediction would be [25].

## 3. Results

The results of the predictive performance of the fitted Cox models based on approximation wavelet coefficients, the top number of principal components, and partial least squares components for simulated, DLBCL, and lung datasets are shown in Tables 1, 2, and 3, respectively. In general, the results showed that the Cox model based on supervised wavelet feature extraction method was superior to the supervised principal components and partial least squares components in terms of different evaluation criteria for three data sets. Although, in simulated data set, all methods have a similar performance in terms of the Integrated Brier Score.

The results showed that the spread of mean values of five evaluation measures over the 50 data sets is fairly large. These variations are caused by selecting the data at random into 50 data sets as well as the variations of the prediction methods performance for the given datasets. In order to determine how much of the variation was due to the prediction methods, we used the supervised wavelet method as a benchmark and, for each of the two other methods, computed the difference between the evaluation criteria in each of the conditions.

**Figure 2:**
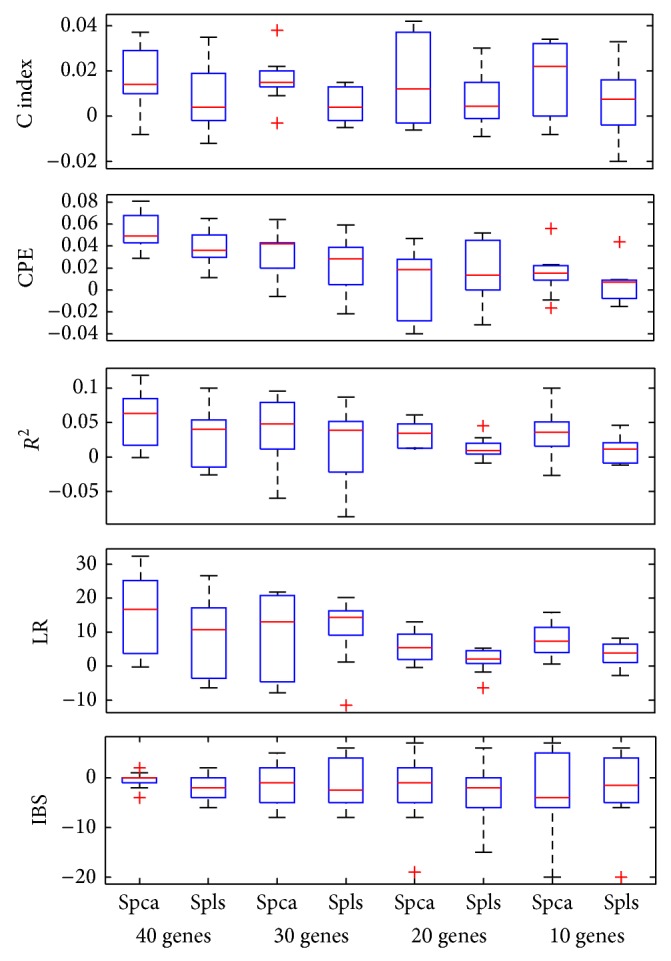
Box plot of the difference in model evaluation criteria between the supervised wavelet and the two other methods for simulated dataset with different number of preselected genes.

**Figure 3:**
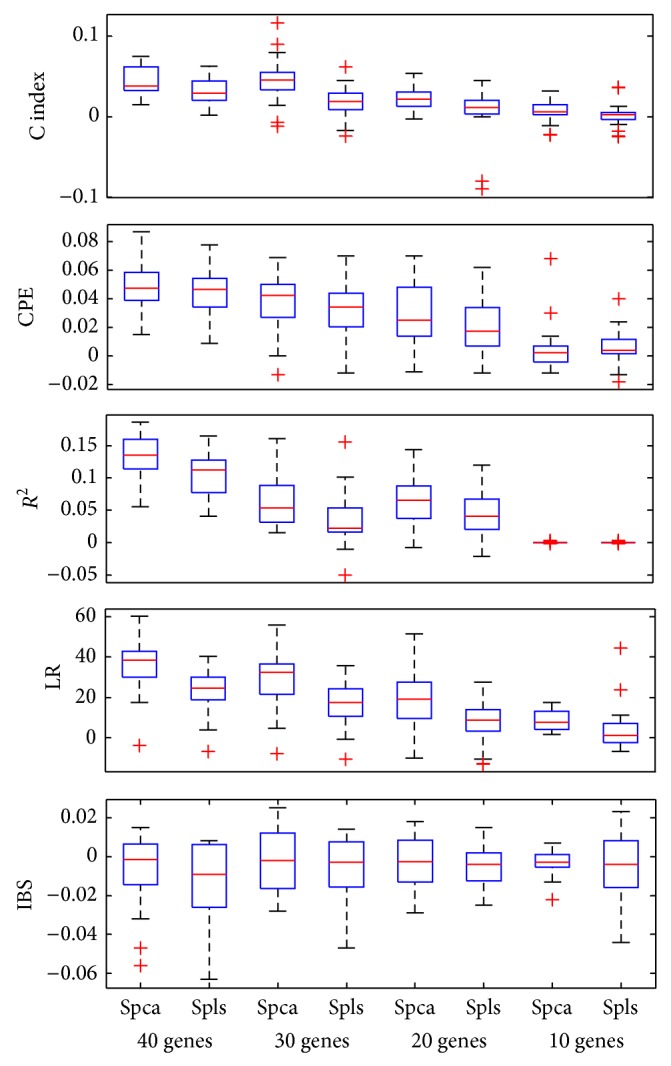
Box plot of the difference in model evaluation criteria between the supervised wavelet and the two other methods for DLBCL dataset with different number of preselected genes.

**Figure 4:**
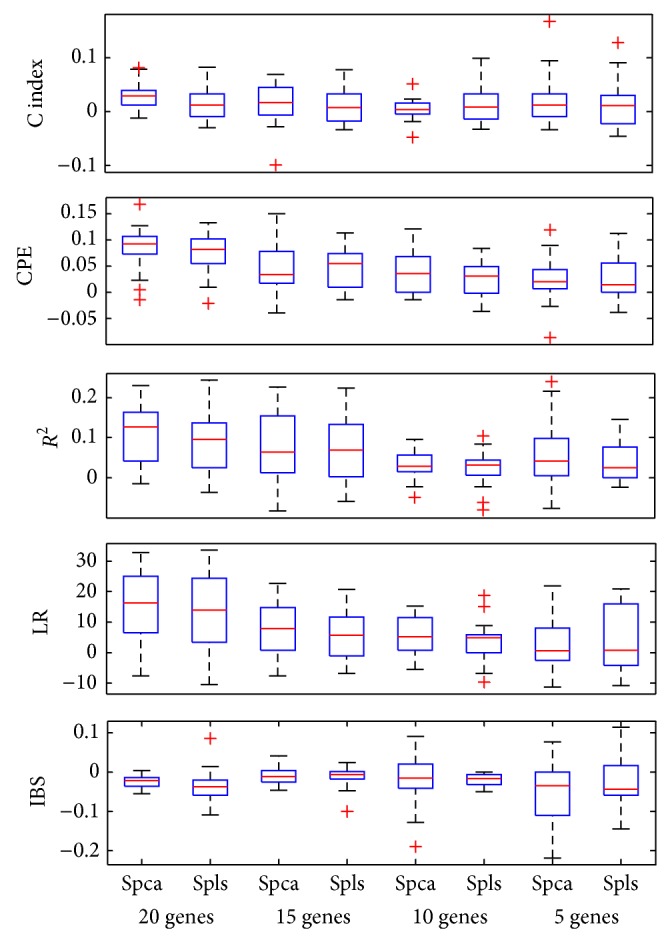
Box plot of the difference in model evaluation criteria between the supervised wavelet and the two other methods for Lung dataset with different number of preselected genes.

Figures 2, 3, and 4 showed the boxplots of these differences in each evaluation criterion for the 50 data sets. The median values for *R* ^2^, C index, CPE, and LR were positive, which showed that supervised wavelet method performed better than other methods. In addition, the median values for the Integrated Brier Score criterion in the different conditions were negative. Totally, simulation results and real data analysis confirmed the suitable performance of the supervised wavelet method.

The results of the predictive performance of the fitted Cox models based on combination of clinical and genomic information for a lung data set are shown in Table 4. The results showed that adding clinical information leads to an increase in the predictive ability of the model in three mentioned methods (supervised wavelet, supervised PCA, and supervised PLS).

## 4. Discussion

This study employed the supervised dimension reduction method based on wavelet transform and modeled survival times in the presence of right censoring, taking into account the microarray data information. The proposed method was evaluated by simulations and applied to the Rosenwald et al.'s DLBCL dataset and Beer et al.'s Lung cancer dataset [7, 21].

Considering the fact that most genes are irrelevant to patients' survival, we analyzed the reduced dataset given by selecting genes that were significantly related to survival time based on the Wald's statistic. If the wavelet transform is performed directly by using all of the genes in a data set, there is no guarantee that the resulting wavelet coefficients will be related to survival [19, 22]. Thus, this study introduced a supervised form of wavelet transform that can be considered supervised wavelet. After extracting supervised wavelet approximation coefficients using discrete Haar wavelet transform, the coefficients had higher predictive performance than the top number of principal components and the top number of partial least squares components. Hence, our results suggested that the wavelet coefficients are an efficient way to characterize the features of high dimensional microarray data. It seems that these results exhibit the possibility of developing more efficient tools using wavelets for the dimensionally reduction of microarray data sets in the context of survival analysis.

The main purpose of the feature extraction method using wavelet transform is that the approximation coefficients usually comprise the majority of the important information [14]. In addition, this method can usually condense or denoise a signal without appreciable degradation due to using a different view of data than those presented by conventional methods. In addition, the powerful capability of the DWT to compress the signal energy makes it a good candidate for feature extraction applications. The DWT compresses most of the energy from the input signal and concentrates it in a few high-magnitude coefficients in the transformed matrix. The DWT also reduces the size of the input signal to half of its original size. Keeping only a number of these high-magnitude coefficients (in addition to their locations) while discarding the rest of the coefficients in the transformed signal can produce a valid feature vector representation of the input signal [13].

The wavelet feature extraction method does not depend on the training dataset to obtain the basis of feature space compared to PCA and PLS methods. Therefore, the wavelet feature extraction method reduces the computation load compared to PCA and PLS [15].

The flexible characteristic of our proposed method makes it appropriate not only for correlating censored patient survival data with microarray gene expression data but also with large-scale biological data stemming from other high-throughput technologies such as DNA copy number analysis and proteomics.

Although the proposed method was better than supervised principal components and supervised partial least squares components based on two popular data sets and brief simulation, it is suggested that comprehensive simulation is used in future studies in order to evaluate this method compared with the other dimension reduction methods.

The future investigations can focus on different ways of preselecting gene in the first stage of the proposed method. For example, rather than ranking genes based on their Wald's statistic, one would use a different metric to measure the association between a given gene and survival time. On the other hand, another mother wavelet and different level of decomposition can be studied.

## 5. Conclusion

This study showed the Cox model based on supervised wavelet feature extraction method which had superior predictive performance over the supervised principal components and supervised partial least squares components based on top selected genes. These results exhibit the possibility of developing more advanced tools using wavelets for the dimension reduction of microarray data sets in the context of survival analysis.

## Figures and Tables

**Table 1:** Performance of different Cox models for simulated dataset.

# Gene	Method	C index ± se	CPE ± se	*R* ^2^ ± se	LR ± se	IBS ± se
40	Supervised wavelet	0.924 ± 0.002	0.904 ± 0.003	0.766 ± 0.006	96.906 ± 1.729	0.153 ± 0.000
	Supervised PCA	0.907 ± 0.002	0.850 ± 0.003	0.709 ± 0.003	81.564 ± 0.700	0.153 ± 0.000
	Supervised PLS	0.919 ± 0.005	0.865 ± 0.005	0.739 ± 0.005	89.083 ± 1.311	0.155 ± 0.000

30	Supervised wavelet	0.914 ± 0.002	0.877 ± 0.004	0.720 ± 0.009	83.313 ± 2.284	0.150 ± 0.000
	Supervised PCA	0.897 ± 0.003	0.842 ± 0.014	0.684 ± 0.007	76.448 ± 1.410	0.151 ± 0.004
	Supervised PLS	0.910 ± 0.003	0.853 ± 0.016	0.711 ± 0.008	82.436 ± 1.791	0.151 ± 0.004

20	Supervised wavelet	0.899 ± 0.006	0.837 ± 0.030	0.682 ± 0.005	72.253 ± 2.233	0.153 ± 0.003
	Supervised PCA	0.886 ± 0.004	0.827 ± 0.025	0.648 ± 0.009	69.357 ± 1.873	0.154 ± 0.004
	Supervised PLS	0.895 ± 0.003	0.835 ± 0.027	0.669 ± 0.011	73.691 ± 2.273	0.154 ± 0.003

10	Supervised wavelet	0.870 ± 0.006	0.823 ± 0.023	0.618 ± 0.013	65.800 ± 1.419	0.154 ± 0.004
	Supervised PCA	0.855 ± 0.011	0.810 ± 0.002	0.582 ± 0.008	58.072 ± 1.845	0.154 ± 0.003
	Supervised PLS	0.866 ± 0.009	0.818 ± 0.001	0.609 ± 0.009	62.484 ± 1.767	0.156 ± 0.003

**Table 2:** Performance of different Cox models for DLBCL dataset.

# Gene	Method	C index ± se	CPE ± se	*R* ^2^ ± se	LR ± se	IBS ± se
40	Supervised wavelet	0.755 ± 0.005	0.744 ± 0.004	0.401 ± 0.011	78.739 ± 1.815	0.237 ± 0.007
	Supervised PCA	0.711 ± 0.004	0.695 ± 0.003	0.270 ± 0.000	42.636 ± 1.762	0.245 ± 0.005
	Supervised PLS	0.723 ± 0.003	0.698 ± 0.003	0.294 ± 0.007	55.883 ± 1.449	0.250 ± 0.005

30	Supervised wavelet	0.723 ± 0.005	0.727 ± 0.007	0.325 ± 0.013	70.303 ± 2.618	0.244 ± 0.004
	Supervised PCA	0.709 ± 0.004	0.692 ± 0.003	0.262 ± 0.008	42.087 ± 1.825	0.245 ± 0.003
	Supervised PLS	0.713 ± 0.002	0.697 ± 0.002	0.289 ± 0.007	54.898 ± 1.418	0.251 ± 0.004

20	Supervised wavelet	0.730 ± 0.002	0.714 ± 0.002	0.323 ± 0.009	59.708 ± 2.699	0.243 ± 0.004
	Supervised PCA	0.709 ± 0.003	0.688 ± 0.003	0.260 ± 0.008	41.327 ± 2.079	0.245 ± 0.003
	Supervised PLS	0.719 ± 0.002	0.696 ± 0.003	0.282 ± 0.006	53.130 ± 1.486	0.249 ± 0.004

10	Supervised wavelet	0.703 ± 0.004	0.686 ± 0.005	0.255 ± 0.007	49.838 ± 1.832	0.248 ± 0.003
	Supervised PCA	0.699 ± 0.005	0.686 ± 0.003	0.254 ± 0.013	41.056 ± 2.045	0.252 ± 0.004
	Supervised PLS	0.701 ± 0.003	0.684 ± 0.003	0.255 ± 0.007	45.648 ± 2.241	0.254 ± 0.006

**Table 3:** Performance of different Cox models for lung cancer dataset.

# Gene	Method	C index ± se	CPE ± se	*R* ^2^ ± se	LR ± se	IBS ± se
20	Supervised wavelet	0.923 ± 0.005	0.876 ± 0.007	0.582 ± 0.014	54.986 ± 2.130	0.328 ± 0.015
	Supervised PCA	0.892 ± 0.003	0.796 ± 0.010	0.471 ± 0.014	38.609 ± 1.637	0.353 ± 0.009
	Supervised PLS	0.909 ± 0.005	0.801 ± 0.005	0.498 ± 0.008	40.77 ± 1.439	0.365 ± 0.011

15	Supervised wavelet	0.905 ± 0.004	0.846 ± 0.005	0.531 ± 0.007	45.466 ± 1.838	0.343 ± 0.007
	Supervised PCA	0.894 ± 0.003	0.801 ± 0.007	0.469 ± 0.010	38.263 ± 1.678	0.349 ± 0.007
	Supervised PLS	0.900 ± 0.002	0.803 ± 0.005	0.483 ± 0.008	39.954 ± 1.382	0.353 ± 0.009

10	Supervised wavelet	0.889 ± 0.006	0.813 ± 0.006	0.462 ± 0.018	38.357 ± 1.641	0.330 ± 0.010
	Supervised PCA	0.878 ± 0.005	0.784 ± 0.009	0.441 ± 0.008	34.217 ± 1.671	0.335 ± 0.008
	Supervised PLS	0.885 ± 0.003	0.788 ± 0.004	0.448 ± 0.007	36.087 ± 1.356	0.350 ± 0.007

5	Supervised wavelet	0.873 ± 0.006	0.795 ± 0.005	0.429 ± 0.001	31.906 ± 1.786	0.297 ± 0.007
	Supervised PCA	0.853 ± 0.005	0.775 ± 0.006	0.387 ± 0.012	29.241 ± 1.784	0.315 ± 0.006
	Supervised PLS	0.858 ± 0.005	0.771 ± 0.006	0.386 ± 0.010	29.650 ± 1.313	0.323 ± 0.006

**Table 4:** Performance of different Cox models for lung cancer dataset (clinical + genomic data).

# Gene	Method	C index ± se	CPE ± se	*R* ^2^ ± se	LR ± se	IBS ± se
20	Supervised wavelet	0.949 ± 0.006	0.924 ± 0.010	0.669 ± 0.031	72.304 ± 2.589	0.431 ± 0.007
	Supervised PCA	0.907 ± 0.008	0.844 ± 0.009	0.553 ± 0.033	52.020 ± 2.208	0.432 ± 0.007
	Supervised PLS	0.914 ± 0.007	0.849 ± 0.009	0.564 ± 0.035	53.814 ± 2.366	0.435 ± 0.009

15	Supervised wavelet	0.916 ± 0.005	0.855 ± 0.011	0.558 ± 0.031	56.318 ± 3.017	0.433 ± 0.010
	Supervised PCA	0.903 ± 0.007	0.836 ± 0.010	0.540 ± 0.034	53.478 ± 2.585	0.435 ± 0.009
	Supervised PLS	0.908 ± 0.007	0.842 ± 0.012	0.552 ± 0.041	55.526 ± 2.398	0.435 ± 0.006

10	Supervised wavelet	0.906 ± 0.006	0.848 ± 0.008	0.552 ± 0.027	52.746 ± 2.872	0.426 ± 0.006
	Supervised PCA	0.892 ± 0.009	0.831 ± 0.008	0.521 ± 0.029	48.092 ± 2.119	0.426 ± 0.007
	Supervised PLS	0.905 ± 0.009	0.842 ± 0.009	0.542 ± 0.031	51.472 ± 2.562	0.430 ± 0.005

5	Supervised wavelet	0.895 ± 0.008	0.818 ± 0.011	0.499 ± 0.036	51.472 ± 2.760	0.352 ± 0.008
	Supervised PCA	0.883 ± 0.009	0.803 ± 0.010	0.445 ± 0.042	46.336 ± 2.113	0.359 ± 0.008
	Supervised PLS	0.879 ± 0.007	0.814 ± 0.010	0.481 ± 0.029	49.976 ± 2.152	0.355 ± 0.006
